# Phytochrome-induced *SIG2* expression contributes to photoregulation of phytochrome signalling and photomorphogenesis in *Arabidopsis thaliana*

**DOI:** 10.1093/jxb/ert308

**Published:** 2013-09-27

**Authors:** Sookyung Oh, Beronda L. Montgomery

**Affiliations:** ^1^Department of Energy—Plant Research Laboratory, Michigan State University, East Lansing, MI 48824, USA; ^2^Department of Biochemistry and Molecular Biology, Michigan State University, East Lansing, MI 48824, USA

**Keywords:** Light signalling, nuclear gene expression, plastid gene expression, photomorphogenesis, phytochrome, sigma factor.

## Abstract

Chloroplast-localized sigma factor (SIG) proteins promote specificity of the plastid-encoded RNA polymerase. SIG2 function appears to be necessary for light-grown *Arabidopsis thaliana* plants. Specific photoreceptors or light-dependent factors that impact the light-induced accumulation of SIG2 have not been reported. A molecular link between phytochromes and nuclear-encoded SIG2, which impacts photomorphogenesis specifically under red (R) and far-red (FR) light, is described here. Both phyA and phyB promote *SIG2* transcript accumulation. Disruption of *SIG2* results in R- and FR-specific defects in the inhibition of hypocotyl elongation and cotyledon expansion, although no impairments in these responses are detected for *sig2* mutants under blue (B) or white (W) light. SIG2 also impacts root elongation under W and R, and the R-dependent expression of *PIF4*, encoding a phytochrome-interacting factor, and *HY2*, which encodes a phytochrome chromophore biosynthetic enzyme. Whereas SIG2 apparently impacts the accumulation of the phytochromobilin (PΦB) phytochrome chromophore, *sig2* mutants differ significantly from PΦB mutants, primarily due to wavelength-specific defects in photomorphogenesis and disruption of a distinct subset of phytochrome-dependent responses. The molecular link between phytochromes and SIG2 is likely to be an important part of the co-ordination of gene expression to maintain stoichiometry between the nuclear-encoded phytochrome apoprotein and plastid-derived PΦB, which combine to form photoactive phytochromes, and/or light-dependent SIG2 accumulation is involved in an inductive light signalling pathway co-ordinating components between nucleus and plastids.

## Introduction

The establishment of photosynthesis during plant development requires the assembly of functional chloroplasts, which is a light-regulated process requiring fine co-ordination of the expression of nuclear- and chloroplast-encoded genes. The major photosynthetic enzyme Rubisco is composed of a small subunit RbcS, which is encoded in the nuclear genome, and the chloroplast genome-encoded large subunit RbcL. The assembly of Rubisco has often been used as a model for understanding the mechanism(s) by which plants maintain stoichiometry between nuclear- and plastid-encoded components (Rodermel, 1999; Wostrikoff and Stern, 2007; Suzuki and Makino, 2012). The involvement of photoreceptors in regulating the expression of a number of nuclear-encoded genes required for chloroplast development has been reported (Tyagi and Gaur, 2003; Larkin and Ruckle, 2008). In recent studies investigating the function of mesophyll-localized phytochromes, it was noted that both RbcL and RbcS protein levels were reduced in lines depleted of phytochromes, which suggested a distinct role for phytochromes in co-ordinating the stoichiometry of nuclear- and plastid-derived components of Rubisco (Oh and Montgomery, 2011).

Plant photoreceptors mediate a number of light-dependent growth and developmental responses, including seed germination, inhibition of hypocotyl elongation, chloroplast development, cotyledon and leaf expansion, promotion of root elongation, and the photoperiodic induction of flowering, among others (Franklin and Quail, 2010; Kami *et al.*, 2010). Phytochromes are photoreceptors that consist of two distinct components, i.e. a nuclear-encoded apoprotein and a plastid-synthesized linear tetrapyrrole chromophore. There are five nuclear-encoded genes for phytochrome apoproteins in Arabidopsis, i.e. *PHYA* to *PHYE* (Sharrock and Quail, 1989; Quail, 1994). A single chromophore exists, i.e. phytochromobilin (PΦB), which is synthesized fully in the plastid and exported to the cytosol, where it becomes covalently attached to all of the apoproteins to produce photoactive holophytochromes (Terry *et al.*, 1993).

Among the phytochrome family members, phyA is primarily responsible for perceiving far-red (FR) light (Nagatani *et al.*, 1993; Parks and Quail, 1993; Whitelam *et al.*, 1993), although it also functions in sensing blue (B) light (Neff and Chory, 1998; Poppe *et al.*, 1998; Whitelam *et al.*, 1998; Chun *et al.*, 2001; Weller *et al.*, 2001; Duek and Fankhauser, 2003; Yadav *et al.*, 2005; Stephenson and Terry, 2008; Warnasooriya *et al.*, 2011) and high irradiance red (R) light (Franklin *et al.*, 2007). The remaining four phytochromes, i.e. phyB to phyE, primarily contribute to the regulation of photomorphogenesis in response to perceiving R light (Nagatani *et al.*, 1993; Reed *et al.*, 1993; Aukerman *et al.*, 1997; Devlin et al., 1998, 1999; Franklin *et al.*, 2003; Monte *et al.*, 2003; Warnasooriya *et al.*, 2011). Phytochromes can function as homodimers (Jones and Quail, 1986; Wagner *et al.*, 1996; Sharrock and Clack, 2004; Clack *et al.*, 2009) or, in some cases have been reported to function as obligate heterodimers (Sharrock and Clack, 2004; Clack *et al.*, 2009).

A number of plastid-encoded genes involved in photosynthesis are transcribed by the plastid-encoded RNA polymerase (PEP), whose activity can be regulated by nuclear-encoded sigma (SIG) factor proteins (Kanamuru *et al*, 1999). SIG proteins are promoter-specificity factors that function with PEP in *Arabidopsis* (Hanaoka *et al.*, 2003). There are six such proteins (SIG1 to SIG6) in *Arabidopsis* (Allison, 2000). SIG2 (Shirano *et al.*, 2000) and SIG6 (Ishizaki *et al.*, 2005) have been implicated in the regulation of chlorophyll synthesis and/or accumulation in *Arabidopsis*.

Expression of *SIG2* was previously shown to be green-tissue- or leaf-specific (Isono *et al.*, 1997), as well as light-induced in *Arabidopsis* (Isono *et al.*, 1997; Privat *et al.*, 2003). Prior studies also showed that SIG2 is a chloroplast-localized protein (Kanamaru *et al.*, 1999; Woodson *et al.*, 2013). Light-grown *sig2* mutants have a pale-green phenotype and abnormal plastids, whereas normal etioplasts form in the mutants grown in the dark (Shirano *et al.*, 2000; Kanamaru *et al.*, 2001). In this regard, the SIG2 function appears to be light dependent. Tetrapyrrole synthesis and accumulation are impaired in the *sig2-2* mutant with reduced levels of several tetrapyrroles, including δ-aminolevulinic acid (ALA), protochlorophyllide, chlorophyll, and haem (Woodson *et al.*, 2013). Mutation of *SIG2* is associated with reduced glutamyl-tRNA (tRNA^Glu^) levels (Kanamaru *et al.*, 2001; Hanaoka *et al.*, 2003; Woodson *et al.*, 2013), which results in the reported reduced levels of tetrapyrroles in *sig2* mutants.

A SIG2-dependent link between the plastid and nuclear genome has been previously reported (Shirano *et al.*, 2000). Recent work indicated that a *sig2* mutant is impacted in plastid transcription and retrograde signalling from the plastid to the nucleus that co-ordinates gene expression between these two organelles in response to the functional status of plastids (Woodson *et al.*, 2013). Relatedly, *SIG2* is expressed early in development (Ishizaki *et al.*, 2005), suggesting important roles during the light-dependent establishment of seedlings. One such role could be in co-ordinating gene expression to maintain stoichiometry of nuclear- and plastid-encoded components in light-grown plants, e.g. nuclear-encoded apoproteins and chloroplast-synthesized chromophores of tetrapyrrole-containing proteins. A previous study indicated a link between photoreceptors cry1 and cry2 and B light-dependent regulation of SIG5 (Onda *et al.*, 2008). *SIG5* expression had been shown previously to be particularly responsive to B light, and SIG5 shown to function in induction of *photosystem II reaction center protein D* (*psbD*) (Tsunoyama *et al.*, 2002). The specific photoreceptors or light-dependent factors that impact light-induced accumulation of other SIG proteins have not been reported.

Here, we describe a molecular link between phytochromes and SIG2, which impacts distinct aspects of photomorphogenesis in *Arabidopsis*. The role of phytochromes in regulating the induction of *SIG2* may be an important part of maintaining the balance between the nuclear-encoded phytochrome apoprotein and the plastid-derived PΦB chromophore, which combine to form photoactive phytochrome photoreceptors, and/or a component of anterograde signalling, i.e. forward communication from the nucleus that controls plastid development and signalling. In the latter regard, SIG2 may function both in retrograde signalling from the plastid to the nucleus (Woodson *et al.*, 2013), as well as in an inductive light signalling pathway between the nucleus and plastids.

## Materials and methods

### Plant materials

*A. thaliana* WT Col-0 ecotype, T-DNA insertional mutant lines *sig2-2* (SALK_045706; Kanamaru *et al.*, 2001; Woodson *et al.*, 2013), *sig2-3* (SALK_022546), *sig6-1* (SAIL_893_C09; Woodson *et al.*, 2013), *hy5* (SALK_056405), and *pif4* (SALK_140393) were obtained from the *Arabidopsis* Biological Resource Center (ABRC; http://www.biosci.ohio-state.edu/pcmb/Facilities/abrc/abrchome.htm, last accessed 3 September 2013; Alonso *et al.*, 2003). No-0 WT and transgenic *BVR* (i.e. 35S::pBVR3 and CAB3::pBVR2) lines have been described previously (Montgomery *et al.*, 1999; Warnasooriya and Montgomery, 2009). All mutant genotypes for SALK lines were confirmed by PCR using primers designed with the web-based SALK T-DNA Primer Design tool (http://signal.salk.edu/tdnaprimers.2.html, last accessed 3 September 2013) and primer sequences are listed in Table 1. Confirmed homozygous mutant plants were used in this study. T-DNA insertional mutants *phyA* (SALK_014575; Mayfield *et al.*, 2007; Ruckle *et al.*, 2007) and *phyB* (SALK_022035; Mayfield *et al.*, 2007; Ruckle *et al.*, 2007) used in these studies have previously been described; whereas the double mutant *phyAphyB* was obtained from a genetic cross between the two single mutants.

**Table 1.** Primers used in this study

AGI number	LP or forward primer sequence (5′–3′)	RP or reverse primer sequence (5′–3′)	Purpose
*At1g08540* (*SIG2*)			*sig2-2* (SALK_045706)
	CTTCTTCGTCTTCATCATCCG	CATTGAAGATTTGGGAACCAC	T-DNA mutant screening
			*sig2-3* (SALK_022546)
	GTACAGTTGCTTTCGTGCCTC	GAGAAGAACATGAGCTGTCGG	T-DNA mutant screening
	TGTCGGCAGGAATACAGGACCTT	TCCGATTTTCTGGTCTAGCGACCTC	RT-PCR analysis
	AGAGGCACGAAAGCAACTGTA	AGGAGAGAGTAGAACCGCCAT	qRT-PCR analysis
*At1g08550* (*NPQ1*)			*npq1-3* (SALK_124757)
	CATCTGCAGATGCAGTTGATG	CATCTGCAGATGCAGTTGATG	T-DNA mutant screening
	CTTGCGCGTTCCTTATTGTTCCA	TTGCACGAATTTTTGTACGGCTGA	RT-PCR analysis
*At2g43010* (*PIF4*)	CAGCCGATGGAGATGTTGAGA	TAGTGGTCCAAACGAGAACCG	qRT-PCR analysis
*At2g26670* (*HY*1)	TTCTACAAATGGGACGGCGAA	TAGTCCACTCCTCTGCAACCT	qRT-PCR analysis
*At3g09150* (*HY2*)	ATCAGTTGACTGACCAGACGG	CTCCCCATGGGAAAGTCTCAG	qRT-PCR analysis
*At5g25760* (*UBC21*)	CCTTACGAAGGCGGTGTTTTTCAG	CGGCGAGGCGTGTATACATTTG	RT-PCR analysis
	CAAATGGACCGCTCTTATCAAAG	CTGAAAAACACCGCCTTCGT	qRT-PCR analysis

### Additional mutant allele for SIG2

*Arabidopsis* transgenic line *sig2-4* was used as an additional mutant allele for *SIG2*. *sig2-4* was a line derived from *NPQ1* complementation of the *sig2-4npq1-3* mutant (SALK_124757). pVC1, which contained a WT copy of *NPQ1* in vector pBIN19 (a gift from Dr Niyogi) was used to complement SALK_124757. A T_3_ single copy, homozygous complementation line was used in this study.

### Light sources

Light sources for far-red (FR; λ_max_~735nm), red (R; λ_max_~670nm), blue (B; λ_max_~470nm), and white (W) light were described previously by Warnasooriya and Montgomery (2009). Fluence rates of R, B, and W were measured using a LI-250A Light Meter (LI-COR) connected to a Li-Cor quantum sensor. Fluence rates for FR were measured using a StellarNet EPP2000 spectroradiometer (Apogee Instruments).

### Reverse transcriptase PCR (RT-PCR) analyses

Total RNA was extracted from 7-d-old whole seedlings grown at 22 °C in a growth chamber with continuous FR (FRc) illumination (2.5 or 5 µmol m^–2^ s^–1^) or continuous R (Rc) illumination (50 µmol m^–2^ s^–1^) using the RNeasy^®^ Plant Minikit (Qiagen, CA). cDNA was synthesized using a Reverse Transcription System (Promega, WI) following the instructions of the manufacturer. The first-strand cDNA was used as the template in PCR reactions performed with GoTaqGreen (Promega, WI). Ubiquitin-conjugating enzyme 21 (*UBC21*) was used as an internal control gene, and oligonucleotides were designed using AtRTPrimer (Han and Kim, 2006). The following standard thermal profile was used for PCR: (i) 1 cycle of denaturation at 94 °C for 3min; (ii) 27–30 cycles of denaturation at 94 °C for 30 s, annealing at 58 °C for 30 s, extension at 72 °C for 30 s; and (iii) final extension at 72 °C for 5min. PCR products were visualized using 2% (w/v) agarose gel electrophoresis with ethidium bromide staining. The primers used for RT-PCR are listed in Table 1 and also in Supplementary Table S1 at *JXB* online.

For quantitative RT-PCR (qRT-PCR), cDNA was synthesized using 100ng of total RNA and random primers as described above and the cDNA was mixed with Fast SYBR^®^ Green Master Mix (Applied Biosystems). qPCR was performed using an ABI 7500 Fast Real-Time PCR System (Applied Biosystems) in three technical and three biological replicates. *UBC21* was used as a standard for normalization for *SIG2* in the experiments. The primers used for qRT-PCR are indicated in Table 1 and also in Supplementary Table S1 at *JXB* online.

### Hypocotyl length and cotyledon area measurements

Seeds were surface-sterilized using 35% (v/v) bleach solution and germinated on Murashige and Skoog (MS) media containing 1% (w/v) sucrose adjusted to pH 5.7 with KOH and 0.7% (w/v) Phytoblend agar (Caisson Labs, UT). After cold-stratification at 4 °C for 4 d in darkness, plates were transferred to a growth chamber with appropriate light conditions at 22 °C for 7 d. To quantify hypocotyl lengths, seedlings were scanned and measured using Image J software (NIH). Cotyledons were detached from each seedling, scanned, and cotyledon area measured using Image J software (NIH).

### Root length measurements

Seedlings were grown vertically on MS medium containing 1% (w/v) Suc, 0.05% (w/v) MES, 1% (w/v) Phytoblend agar (Caisson Labs, UT) for 7 d under appropriate light conditions. To quantify root lengths, seedlings were scanned and measured using Image J software (NIH).

## Results

### Expression of SIG2 in phytochrome-inactivation lines

It was demonstrated previously that the hypocotyls of the CAB3::pBVR2 line, which exhibits mesophyll-specific inactivation of phytochromes due to CAB-driven expression of the chromophore-degrading enzyme biliverdin reductase (BVR), are longer than the hypocotyls of No-0 wild-type (WT) and a 35S::pBVR3 line exhibiting constitutive inactivation of phytochromes under continuous FR (FRc) (Warnasooriya and Montgomery, 2009). These results support a role for mesophyll-specific phytochromes in the inhibition of hypocotyl elongation. The more severe phenotype of the CAB3::pBVR2 line under FRc relative to a constitutive *BVR* expression line, i.e. 35S::pBVR3, has previously been attributed to differences in the pattern of BVR accumulation and the associated degradation of the phytochrome chromophore precursor biliverdin in cotyledons of these distinct lines (Oh *et al.*, 2013). It is well established that 35S-driven gene expression is vascular enriched in transgenic plants (Sunilkumar *et al.*, 2002; Hraška *et al.*, 2008); however, CAB3 promoter-driven expression is widespread specifically in mesophyll cells of light-grown plants (Mitra *et al.*, 1989).

**Fig. 1.**
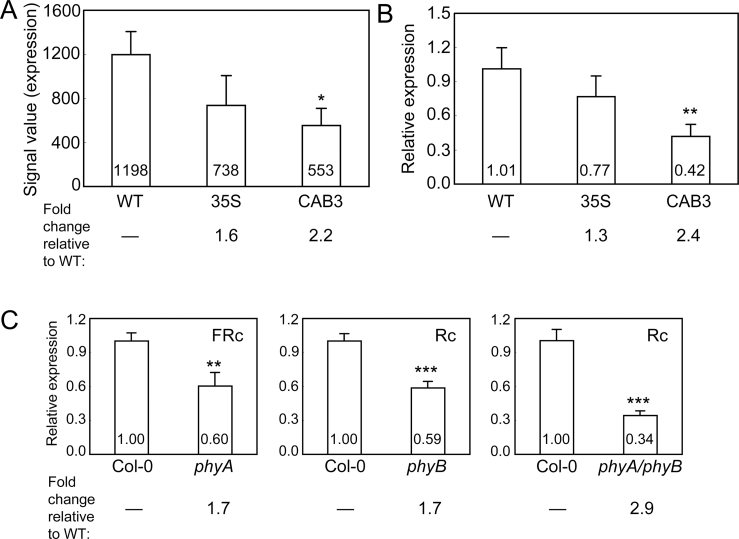
Expression of *SIG2* in phytochrome-inactivation lines. (A) Using microarray data (Rosa *et al.*, 2010; Oh *et al.*, 2013), expression levels (signal value) of *SIG2* in No-0 wild-type (WT), 35S::pBVR3 (35S), and CAB3::pBVR2 (CAB3) are shown (±SD, *n*=3). Signal value indicates signal intensity on the ATH1 array as calculated by the Affymetrix Microarray Suite (MAS). (B) Validation of microarray data for *SIG2* using quantitative RT-PCR (qRT-PCR) analysis. Relative *SIG2* expression level compared with *UBC21* is shown (±SD, *n*=3). (C) qRT-PCR analysis of *SIG2* expression in Col-0 WT, *phyA* (SALK_014575), *phyB* (SALK_022035), or *phyAphyB* double mutant seedlings. Relative *SIG2* expression level compared to *UBC21* is shown (±SD, *n*=3). For all experiments, 7-d-old seedlings were grown on MS medium containing 1% Suc and 0.7% Phytoblend agar at 22 °C under continuous far-red (FRc; 5 µmol m^–2^ s^–1^) or continuous red (Rc; 50 µmol m^–2^ s^–1^). Conditions were FRc for (A) and (B) or as indicated for (C). Unpaired, two-tailed Student’s *t*-test comparing BVR lines or mutant to WT, *P <0.05, **P <0.01, ***P <0.005.

From a microarray-based comparison of CAB3::pBVR2, 35S::pBVR3, and No-0 WT lines grown in FRc light (Rosa *et al.*, 2010; Oh *et al.*, 2013), it was found that, among other genes, some of which encode chloroplast-localized and photosynthesis-related proteins and those impacting light-dependent hypocotyl elongation (Oh *et al.*, 2013), *SIG2* was misregulated. *SIG2* (*At1g08540*), which encodes nucleus-encoded sigma factor 2 for the plastid-encoded RNA polymerase (PEP), was down-regulated by 2.2-fold in CAB3::pBVR2 compared with WT (Fig. 1A). Using qRT-PCR analyses, the down-regulation of *SIG2* was validated in lines exhibiting BVR-dependent inactivation of phytochromes grown under FRc (Fig. 1B). Notably, this misregulation of *SIG2* expression was light-dependent, as *SIG2* mRNA levels were not appreciably down-regulated in dark-grown BVR lines (see Supplementary Fig. S1A at *JXB* online).

### Regulation of SIG2 by phytochromes is light-dependent

As holophytochrome levels are correlated with *SIG2* expression, it was tested whether the expression of *SIG2* is regulated by phytochrome A- and/or B-mediated light signalling pathways. The expression of *SIG2* in *phyA*, *phyB*, or *phyAphyB* mutants was examined in dark- and light-grown seedlings. The levels of *SIG2* mRNA accumulation in dark-grown phytochrome-deficient lines was ~90% of WT levels for single *phy* mutants, and nearly 80% of WT for the *phyAphyB* double mutant (see Supplementary Fig. S1B at *JXB* online), suggesting a minimal impact on *SIG* mRNA accumulation in darkness. Using qRT-PCR analyses, it was shown that *SIG2* mRNA accumulation was reduced in light-grown *phyA*, *phyB*, and *phyAphyB* mutants (Fig. 1C). Notably, down-regulation of *SIG2* was most prominent in the *phyAphyB* double mutant, i.e. down by ~3-fold, compared with single mutants (Fig. 1C). These data suggested that both phyA and phyB contribute to positive, light-dependent transcriptional regulation of *SIG2*.

### Expression of SIG2 in different tissues and light conditions

**Fig. 2.**
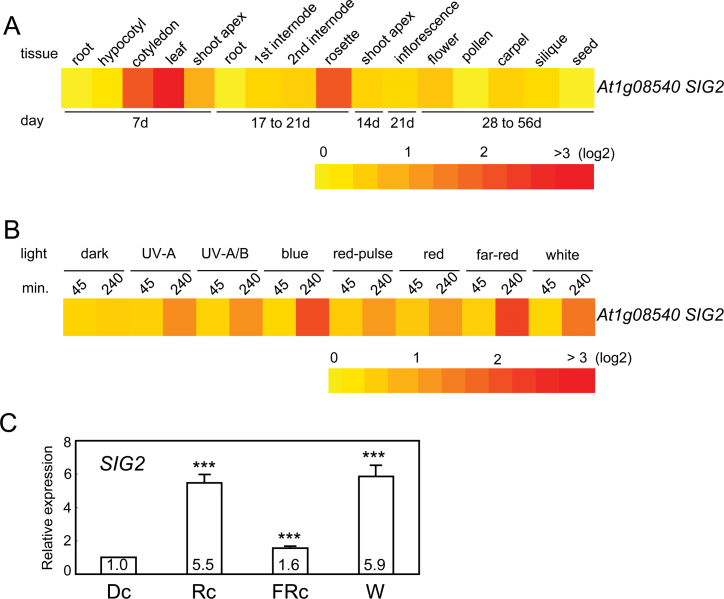
Expression of *SIG2* in different tissues and light conditions. Heat map showing the expression of *SIG2* in different tissues (A) or different light conditions (B) for *Arabidopsis*. For heat map, mean-normalized values of Col-0 WT from AtGenExpress expression library (www.weigelworld.org, last accessed 3 September 2013) and BAR Heatmapper *Plus* (bar.utoronto.ca) were used. For light experiments, aerial parts (hypocotyl and cotyledons) of 4-d-old Col-0 WT seedling grown on MS medium were treated with different light for either 45min or 240min. (C) qRT-PCR analysis of *SIG2* expression in Col-0 WT grown on MS medium containing 1% Suc and 0.7% Phytoblend agar at 22 °C for 7 d under continuous darkness (Dc), continuous red (Rc; 50 µmol m^–2^ s^–1^), continuous far-red (FRc; 5 µmol m^–2^ s^–1^), or white (W; 100 µmol m^–2^ s^–1^). Relative *SIG2* expression level compared with *UBC21* is shown (±SD, *n*=3). Unpaired, two-tailed Student’s *t*-test comparing Rc, FRc, or W to Dc, ***P <0.005.

The expression of *SIG2* was analyzed in different tissues and light conditions using public microarray data for Col-0 WT from AtGenExpress (http://www.weigelworld.org/resources/microarray/AtGenExpress, last accessed 3 September 2013) (Fig. 2). *SIG2* is highly expressed in cotyledon and young leaf tissues from seedlings, as well as in the rosette leaves of adult plants (Fig. 2A). This observation corresponds with prior studies in which *SIG2* expression was shown to be green-tissue- or leaf-specific (Isono *et al.*, 1997) and chloroplast-specific (Kanamaru *et al.*, 1999; Woodson *et al.*, 2013). The public microarray data analysis from AtGenExpress showed the up-regulation of *SIG2* after exposure to 4h of light treatment, including B (10 µmol m^–2^ s^–1^), R (10 µmol m^–2^ s^–1^), FR (10 µmol m^–2^ s^–1^), and W (10 µmol m^–2^ s^–1^) (Fig. 2B), which corresponds to prior observations that *SIG2* expression is light-induced (Isono *et al.*, 1997; Privat *et al.*, 2003). Our qRT-PCR analyses confirmed that *SIG2* expression is light responsive (Fig. 2C). Expression of *SIG2* was up-regulated to a greater degree in response to continuous R (Rc; 50 µmol m^–2^ s^–1^) or white (W; 100 µmol m^–2^ s^–1^) compared with FRc (5 µmol m^–2^ s^–1^) in 7-d-old seedlings (Fig. 2C).

### Identification of sig2 mutants

**Fig. 3.**
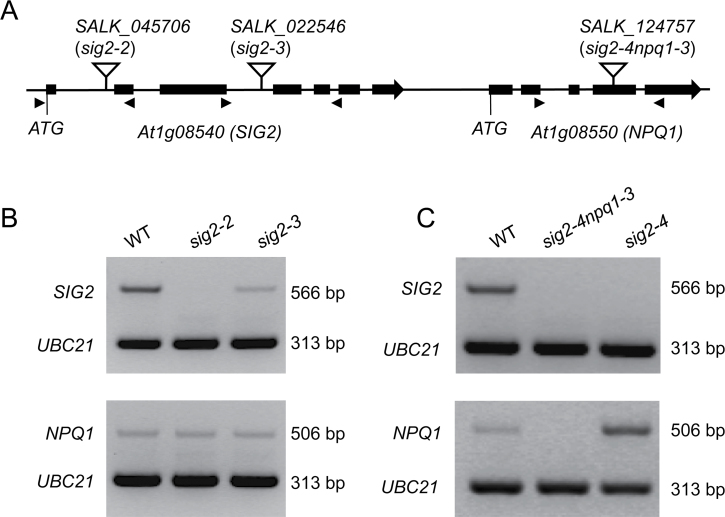
Identification and expression of *SIG2* in SALK T-DNA mutant lines. (A) Schematic representation of the T-DNA insertion sites (inverted open triangle) on map of genomic region containing *SIG2* and its neighbouring gene, *NPQ1*. Black boxes denoted exons. Arrowheads flanking T-DNA insertional sites indicate left primers (LP, ►) and right primers (RP, ◄) used for confirmation of homozygous T-DNA lines. (B) RT-PCR analysis of *SIG2* expression using homozygous SALK T-DNA mutant lines. Seven-day-old Col-0 wild-type (WT) and *sig2* mutant seedlings grown on MS medium containing 1% Suc and 0.7% Phytoblend agar at 22 °C under FRc (2.5 µmol m^–2^ s^–1^) were used. The *UBC21* gene was used as an internal control. As an additional control, the expression of *NPQ1* (a neighbouring gene of *SIG2*) has been tested. Results shown are representative of at least two independent biological replicates. (C) A T_3_ transgenic line with complementation of the *npq1-3* mutation in SALK_124757 (*sig2-4npq1-3*) was used to obtain an additional mutant allele for *SIG2*, which was denoted as *sig2-4*. To validate complementation, the expression of *NPQ1* and *SIG2* was tested using RT-PCR analysis. Seven-day-old Col-0 wild-type (WT) and T_3_ complementation lines grown on MS medium containing 1% Suc and 0.7% Phytoblend agar at 22 °C under FRc (5 µmol m^–2^ s^–1^) were used. The *UBC21* gene was used as an internal control.

Two homozygous SALK T-DNA mutant lines for *SIG2* (*sig2-2* and *sig2-3*) were identified and the T-DNA insertion site of each mutant line was verified by PCR using a T-DNA left border primer coupled with a *SIG2*-specific primer and a pair of *SIG2*-specific primers downstream and upstream of the insertion (Fig. 3A; for primer sequences see Table 1). *sig2-2* (Woodson *et al.*, 2013) and *sig2-3* have T-DNA insertions in the first and third introns, respectively (Fig. 3A). A reduced level of expression of *SIG2* in the isolated T-DNA mutants was confirmed (Fig. 3B). Notably, *SIG2* was heavily down-regulated in *sig2-2* compared with *sig2-3*, suggesting that the *sig2-2* allele is stronger than the *sig2-3* allele (Fig. 3B).

An additional mutant allele was identified for *SIG2* (Fig. 3C). In an assessment of the SALK mutant line SALK_124757, which contains a T-DNA insertion in gene *At1g08550* (*NON-PHOTOCHEMICAL QUENCHING 1*, *NPQ1*), it was noted that, unlike previously identified *npq1* mutant alleles (e.g. *npq1-2*) that exhibited no obvious change in the cotyledon colour (Niyogi *et al.*, 1998), SALK_124757 showed a pale-green phenotype (data not shown). Noticeably, expression of *SIG2*, in addition to *NPQ1*, was down-regulated in SALK_124757 (Fig. 3C), suggesting a polar effect of the T-DNA in this mutant. Given this observation, *NPQ1* expression was tested in *sig2-2* and *sig2-3*, which was not changed in these mutant alleles (Fig. 3B). SALK_124757 was complemented with a WT copy of the *NPQ1* gene, using a construct previously shown to complement *npq1* mutants fully (Niyogi *et al.*, 1998), and complementation was validated by checking the expression of *NPQ1* and *SIG2* via RT-PCR analysis (Fig. 3C). The confirmed T_3_ transgenic line still exhibited a pale-green phenotype and complete down-regulation of *SIG2* (Fig. 3C), suggesting that the resultant T_3_ transgenic complementation line could be used as an additional mutant allele for *SIG2*. This line was designated as *sig2-4* and it was used in further studies.

### SIG2 contributes to inhibition of hypocotyl elongation and expansion of cotyledons under FRc and Rc illumination

**Fig. 4.**
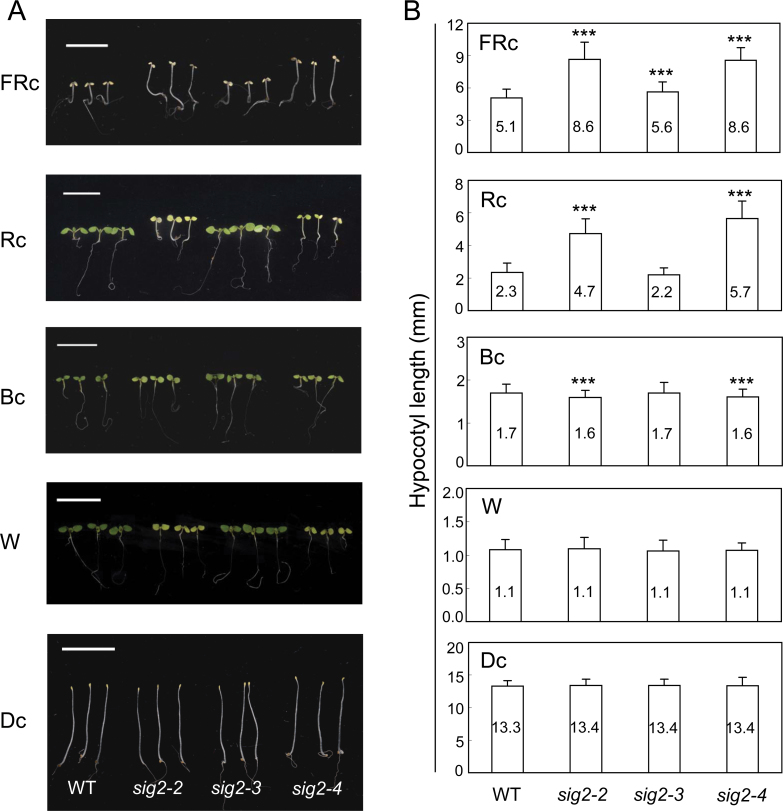
Hypocotyl assay for Col-0 WT and *sig2* mutant seedlings. Seedlings were grown on MS medium containing 1% Suc and 0.7% Phytoblend agar at 22 °C for 7 d under FRc (2.5 µmol m^–2^ s^–1^), Rc (50 µmol m^–2^ s^–1^), Bc (25 µmol m^–2^ s^–1^), W (100 µmol m^–2^ s^–1^) or in darkness (Dc). (A) Representative images of seedlings are shown. Scale bars indicate 1cm. (B) Data points in bar graphs represent mean hypocotyl lengths of seedlings (±SD, *n*=75). Unpaired, two-tailed Student’s *t*-test comparing mutants to WT, ***P <0.005.

As previously reported, a lesion in *SIG2* resulted in pale-green seedlings under light conditions (Fig. 4A; Shirano *et al.*, 2000). Among the tested mutant alleles, *sig2-2* and *sig2-4* exhibited obvious pale-green phenotypes in Rc, continuous B (Bc), and W, whereas *sig2-3* did not (Fig. 4A), supporting the idea that *sig2-2* and *sig2-4* were strong alleles. It was observed that the hypocotyls of strong *sig2* mutant alleles were longer than those of Col-0 WT when plants were grown in FRc or Rc, but not under Bc, W, or in D (Fig. 4). The longer hypocotyl phenotype was most obvious in Rc-grown *sig2-2* and *sig2-4* (Fig. 4; greater than 2-fold), which may correspond to a greater expression of *SIG2* under Rc, when compared with FRc (Fig. 2C). However, *sig2-3*, which was considered a weak mutant allele, did not exhibit any significant changes in hypocotyl length under any light condition (Fig. 4B). These data suggest that SIG2 is required for the accurate inhibition of hypocotyl elongation in FRc and Rc, specifically.

**Fig. 5.**
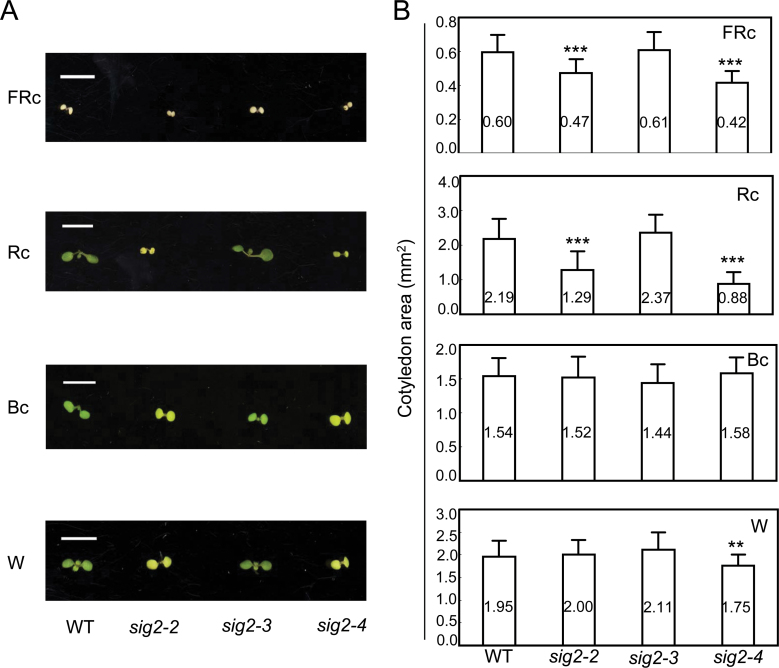
Cotyledon expansion assay for Col-0 WT and *sig2* mutant seedlings. Seedlings were grown on MS medium containing 1% Suc and 0.7% Phytoblend agar at 22 °C for 7 d under FRc (2.5 µmol m^–2^ s^–1^), Rc (50 µmol m^–2^ s^–1^), Bc (25 µmol m^–2^ s^–1^), or W (100 µmol m^–2^ s^–1^). (A) Representative images of cotyledons are shown. Scale bars indicate 0.5cm. (B) Data points in bar graphs represent mean cotyledon area of seedlings (±SD, *n*=34). Unpaired, two-tailed Student’s *t*-test comparing mutants to WT, **P <0.01, ***P <0.005.

In addition to the noted hypocotyl phenotypes, *sig2-2* and *sig2-4* exhibited smaller cotyledons compared with Col-0 WT in FRc or Rc (Fig. 5). However, these mutant alleles did not show changes in cotyledon size when they were grown in Bc or W. The smaller cotyledon phenotype was most obvious in Rc-grown *sig2-2* and *sig2-4* (about 2-fold, Fig. 5B), similar to the more severe hypocotyl elongation phenotype of strong *sig2* mutant alleles under Rc (Fig. 4). *sig2-3* did not exhibit any significant changes in cotyledon size under any light condition tested (Fig. 5). Collectively, our data suggested that SIG2 contributes to the inhibition of hypocotyl elongation and expansion of cotyledons in specific light conditions, namely FRc and Rc.

### SIG2 impacts root elongation under Rc and W illumination

**Fig. 6.**
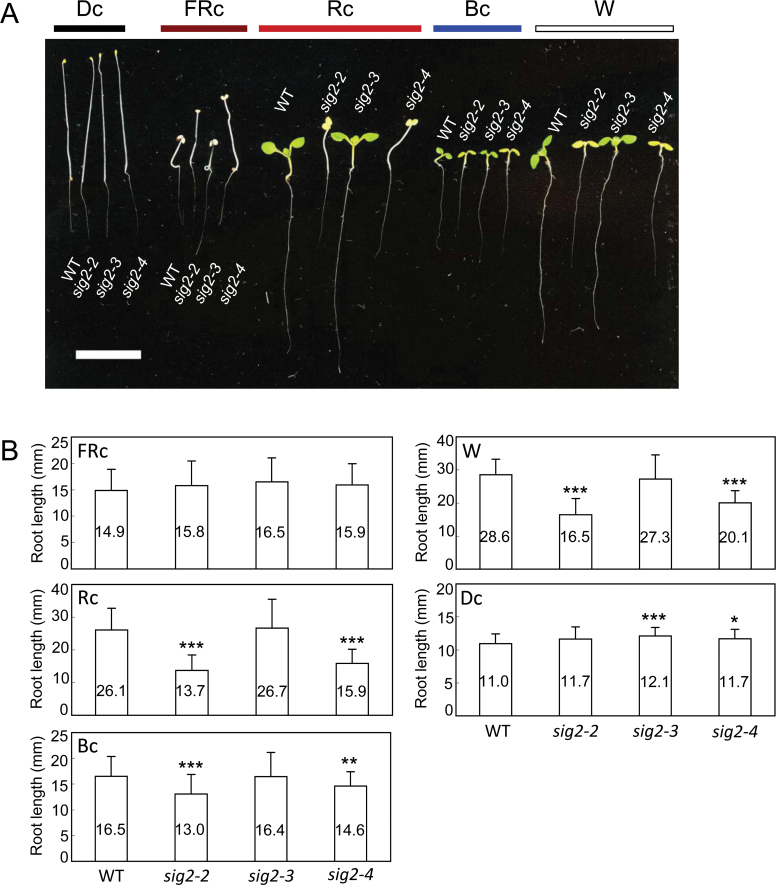
Root assay for Col-0 WT and *sig2* mutant seedlings. Seedlings were grown vertically on MS medium containing 1% Suc, 0.05% MES, and 1% Phytoblend agar at 22 °C for 7 d under FRc (2.5 µmol m^–2^ s^–1^), Rc (50 µmol m^–2^ s^–1^), Bc (25 µmol m^–2^ s^–1^), W (100 µmol m^–2^ s^–1^) or in darkness (Dc). (A) Representative image of seedlings is shown. Scale bar indicates 1cm. (B) Data points in bar graphs represent mean root lengths of seedlings (±SD, *n* ≥34). Unpaired, two-tailed Student’s *t*-test comparing mutants to WT, *P <0.05, **P <0.01, ***P <0.005.

To determine whether other light-dependent phenotypes were observed in *sig2* mutants, root phenotypes were assessed under monochromatic light. In previous studies, it had been demonstrated that both shoot- and root-localized phytochrome-dependent signals can impact the photoregulation of root elongation (Costigan *et al.*, 2011). Although there was no apparent impact on root elongation of *sig2* mutant seedlings in complete darkness compared with WT, strong *sig2* mutants exhibited strongly impaired light-induced elongation of roots relative to WT under Rc and W light conditions, with less severe impacts under Bc (Fig. 6). There was no significant defect observed in the photoregulation of root elongation under FRc for *sig2* mutants when compared with WT (Fig. 6).

### Light-dependent expression of phytochrome genes and phytochrome-signalling component genes in sig2 mutants

The down-regulation of *SIG2* was observed in phytochrome inactivation lines or phytochrome T-DNA mutants (Fig. 1). Therefore, it was investigated whether *SIG2* is required reciprocally for transcriptional regulation of genes encoding phytochromes or some known phytochrome-signalling components. *LONG HYPOCOTYLS 5* (*HY5*), encoding a bZIP transcription factor, contributes to the promotion of photomorphogenesis and is downstream of phytochromes (Oyama *et al.*, 1997; Chattopadhyay *et al.*, 1998). *PHYTOCHROME-INTERACTING FACTOR 4* (*PIF4*) encodes a bHLH factor, which functions as a negative regulator of phytochrome signalling (Huq and Quail, 2002). *LONG HYPOCOTYLS 1* (*HY1*) and *LONG HYPOCOTYLS 2* (*HY2*) encode a haem oxygenase and a ferredoxin-dependent biliverdin reductase, respectively, which are required for the synthesis of the phytochrome chromophore (i.e. phytochromobilin) (Davis *et al.*, 1999; Kohchi *et al.*, 2001; Emborg *et al.*, 2006). Using qRT-PCR analyses, it was determined that the accumulation of *PHYA* and *PHYB* mRNA was reduced by only ~20–30% in strong *sig2* mutants (see Supplementary Fig. S2 at *JXB* online), although the expression of *SIG2* was significantly down (i.e. ~2-fold or greater) in phytochrome-deficient mutants (Fig. 1). For phytochrome signalling components, it was determined that the expression of *HY5*, *PIF4*, *HY1*, or *HY2* genes was not significantly changed in *sig2* mutants grown in FRc for 7 d using RT-PCR analyses (see Supplementary Fig. S3A at *JXB* online). In reciprocal analyses, it was noted that the expression of *SIG2* was not changed in *hy5* or *pif4* mutants grown in FRc for 7 d (see Supplementary Fig. S3B at *JXB* online). These data suggested that, in FRc conditions, these genes are not involved in the *SIG2*-mediated phytochrome-signalling pathway.

**Fig. 7.**
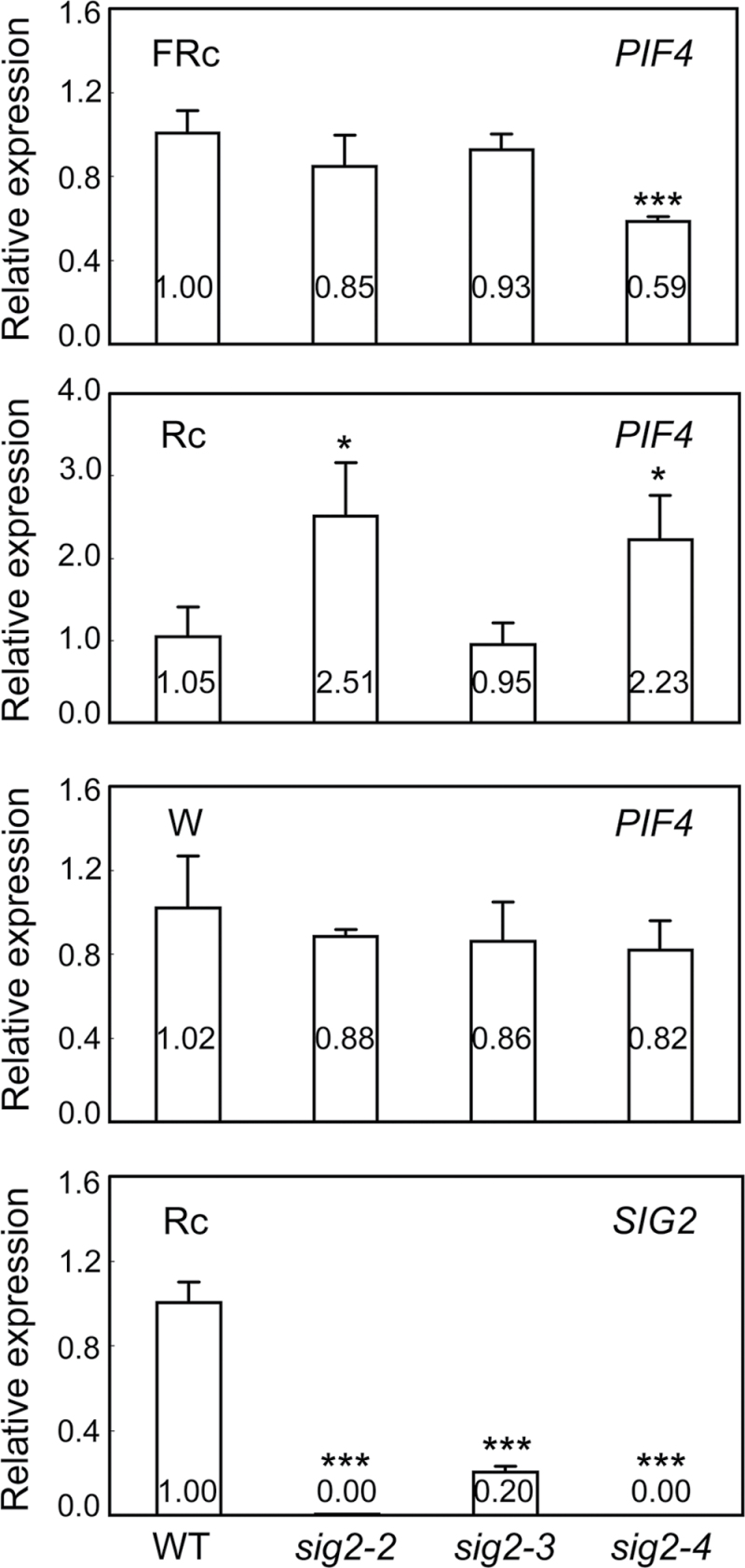
Expression of *PIF4* in Col-0 WT and *sig2* mutant seedlings. Quantitative RT-PCR (qRT-PCR) analyses were performed using 7-d-old Col-0 wild-type (WT) and mutant seedlings grown on MS medium containing 1% Suc and 0.7% Phytoblend agar at 22 °C for 7 d under FRc (2.5 µmol m^–2^ s^–1^), Rc (50 µmol m^–2^ s^–1^), or W (100 µmol m^–2^ s^–1^). Relative gene expression level compared to *UBC21* is shown (±SD, *n*=3). Unpaired, two-tailed Student’s *t*-test comparing mutants to WT, *P <0.05, ***P <0.005.

**Fig. 8.**
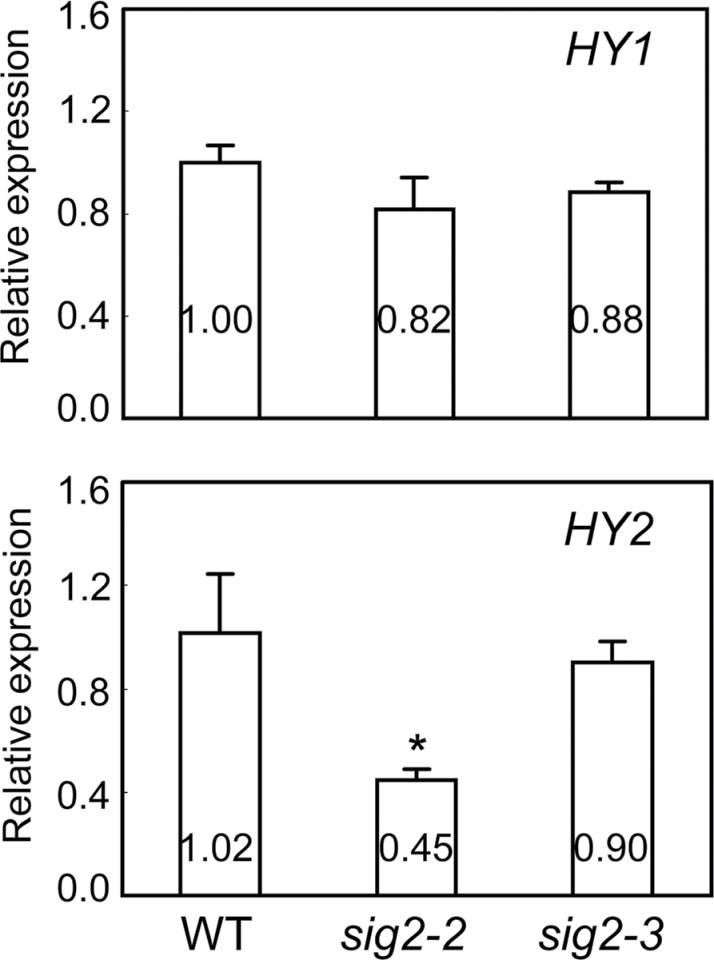
Expression of phytochrome chromophore biosynthesis genes in Col-0 WT and *sig2* mutant seedlings. Quantitative RT-PCR analyses were performed using 7-d-old Col-0 wild-type (WT) and mutant seedlings grown on MS medium containing 1% Suc and 0.7% Phytoblend agar at 22 °C for 7 d under Rc (50 µmol m^–2^ s^–1^). Relative expression level compared to *UBC21* is shown (±SD, *n*=3). Unpaired, two-tailed Student’s *t*-test comparing mutants to WT, *P <0.05.

To test the possibility that the role of *SIG2* in impacting components of the phytochrome-signalling pathway might be wavelength-specific, qRT-PCR of *PIF4* was performed in *sig2* mutants grown in Rc (50 µmol m^–2^ s^–1^), FRc (2.5 µmol m^–2^ s^–1^), and W (100 µmol m^–2^ s^–1^) for 7 d. In Rc, *PIF4* was up-regulated (more than 2-fold) in strong *sig2* mutant alleles (i.e. *sig2-2* and *sig2-4*), whereas no significant change was apparent in a weak *sig2* mutant allele (*sig2-3*) (Fig. 7). However, in FRc or W, the expression of *PIF4* in most *sig2* mutants was comparable with Col-0 WT (Fig. 7). In FRc, a moderate down-regulation of *PIF4* was observed in *sig2-4*. Line *sig2-4* exhibited a great down-regulation of *SIG2* and moderate up-regulation of *NPQ1*, which is a neighbouring gene of *SIG2*, relative to the WT (Fig. 3C). Thus, the observed moderate down-regulation of *PIF4* in FRc-grown *sig2-4* may be caused by ectopic expression of *NPQ1* in FRc-grown *sig2-4* or could be caused by another difference in the *sig2-4* background independent of *SIG2* or *NPQ1* expression. Under Rc, it was also determined that the phytochrome chromophore biosynthesis gene *HY2* was down-regulated more than 2-fold in the strong *sig2* mutant, whereas expression of *HY1* was not significantly impacted (Fig. 8). To address whether the differences in gene expression in FR versus R are due to wavelength-dependent differences in *SIG2* expression in *sig2* mutants, the down-regulation of the *SIG2* gene in Rc-grown *sig2* mutants was confirmed (Fig. 7). Again, no differences were observed in the expression of *SIG2* in *hy5* or *pif4* mutants grown in Rc for 7 d (Fig. S4B).

### SIG6 does not impact the light-dependent inhibition of hypocotyl elongation

**Fig. 9.**
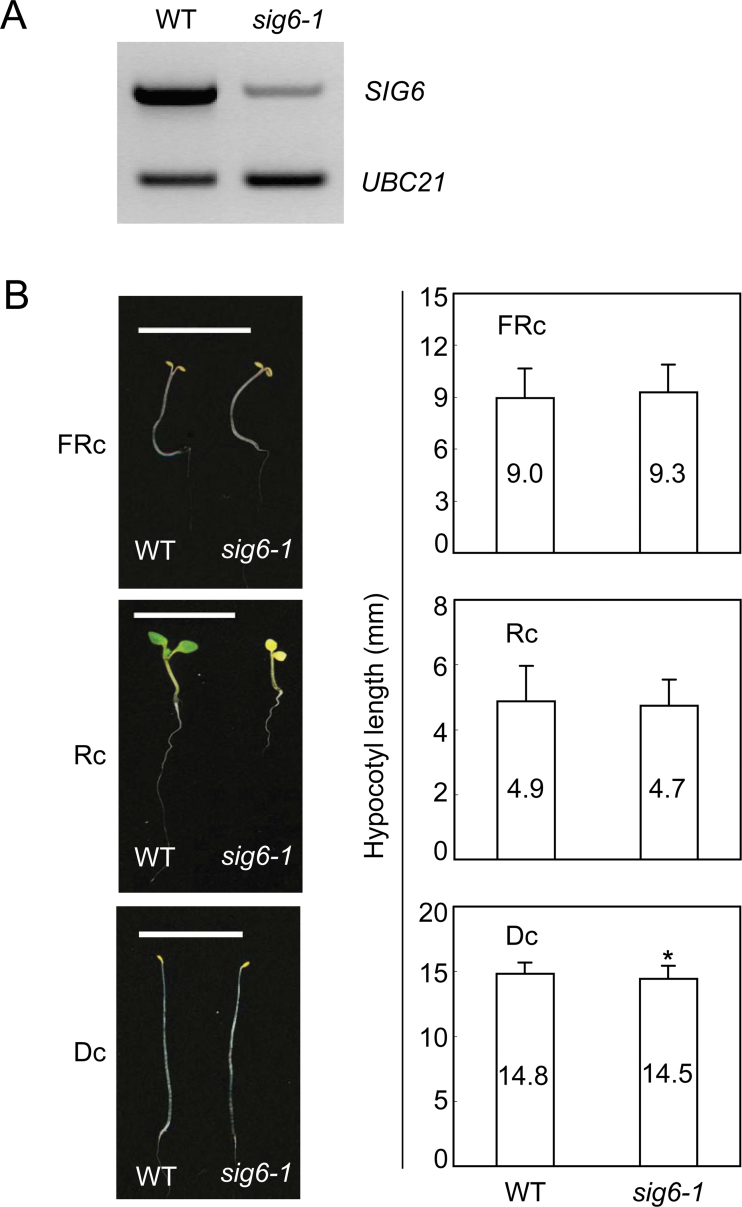
RT-PCR analysis for *SIG6* (*At2g36990*) in *sig6-1* (A) and hypocotyl assay for *sig6-1* (B). Col-0 wild-type (WT) and *sig6-1* (SAIL_893_C09; Woodson *et al.*, 2013) were grown at 22 °C on MS medium containing 1% Suc for 7 d under FRc (2.5 μmol^–2^ s^–1^ Rc (50 µmol m^–2^ s^–1^), or in darkness (Dc). For RT-PCR analysis, the *UBC21* gene was used as an internal control and the results shown were representative of two independent biological replicates. For the hypocotyl assay, the scale bar indicates 1cm and data points in the bar graphs represent mean hypocotyl lengths of seedlings (±SD, *n*=75). Unpaired, two-tailed Student’s *t*-test comparing mutants to WT, *, *P* <0.05.

It was assessed whether the impacts observed for *sig2* mutants were specific to SIG2 function when compared with another SIG protein also shown to impact chloroplast development and plastid transcription, i.e. SIG6 (Ishizaki *et al.*, 2005; Woodson *et al.*, 2013). Both *SIG2* (Shirano *et al.*, 2000) and *SIG6* (Ishizaki *et al.*, 2005) have been implicated in the regulation of chlorophyll synthesis and/or accumulation in *Arabidopsis*. Recent work also indicated that *sig2* and *sig6* mutants are impacted in plastid transcription and retrograde signalling from the plastid to nucleus that co-ordinates gene expression between these two organelles (Woodson *et al.*, 2013). Given these similarities in *SIG2* and *SIG6* function, a *sig6-1* mutant was grown under D, FRc, and Rc conditions in order to determine whether it exhibited light-dependent defects in hypocotyl elongation similar to strong *sig2* mutants. Although the *sig6-1* mutant was severely impaired in SIG6 function as evidenced by the severe chlorophyll deficiency, hypocotyl lengths were not significantly different from WT under any of the conditions that were used to grow the seedlings (Fig. 9). Thus, SIG6 did not have an impact on the regulation of light-dependent hypocotyl elongation, which was in contrast to the FRc- and Rc-dependent hypocotyl elongation observed for strong *sig2* alleles (Fig. 4). These data suggest a distinct role for SIG2 in phytochrome-dependent photomorphogenesis.

## Discussion

Although the role of SIG2 in the regulation of plastid gene expression and retrograde regulation of nuclear-encoded photosynthesis-associated genes is not light dependent, i.e. the expression of Photosynthetic Associated Nuclear Genes (PhANGs) and plastid-encoded transcripts was also down-regulated in dark-grown *sig2* mutant seedlings (Woodson *et al.*, 2013), expression of *SIG2* is impacted by phytochromes (Fig. 1) and light (Fig. 2). Furthermore, SIG2 impacts hypocotyl elongation (Fig. 4) and root elongation (Fig. 6) in a light-dependent manner, i.e. the lengths of hypocotyls or roots of *sig2* mutant seedlings did not differ from WT in complete darkness. Consistent with our observation of light-specific roles for SIG2 in the regulation of growth responses, no phenotype was noted in prior studies of etioplasts in dark-grown *sig2* mutants although plastids were aberrant in light-grown *sig2* seedlings (Shirano *et al.*, 2000; Kanamaru *et al.*, 2001).

Of note, not all phytochrome-dependent phenotypes are disturbed in *sig2* mutants. For example, none of the *sig2* alleles tested differed in the regulation of the expression of a number of known phytochrome biosynthesis or target genes, with the exception of *PIF4* (Fig. 7; see Supplementary Fig. S4 at *JXB* online) and *HY2* (Fig. 8; see Supplementary Fig. S4 at *JXB* online) expression under Rc. Thus, the reduced accumulation of tetrapyrroles in *sig2* mutants (Woodson *et al.*, 2013), which would result in a reduced synthesis of the haem-derived phytochrome chromophore, could be associated specifically with the disruption of the phytochrome-dependent R- and FR-induced inhibition of hypocotyl elongation and cotyledon expansion (Figs 4, 5), R- and W-induced root elongation (Fig. 6), and *PIF4* (Fig. 7) and *HY2* (Fig. 8) expression under Rc. It has been debated previously whether plastid-derived signals may be associated with the phytochrome control of PhANGs (Vinti *et al.*, 2005) or plant development (Møller *et al.*, 2001). In this regard, our results support a role for SIG2 in the promotion of the expression of a plastid-derived gene(s) or protein(s) that impacts expression of *PIF4* and *HY2* in the nucleus.

*SIG2* is expressed early in seedling development (Kanamaru *et al.*, 1999), which may be correlated with a link to phytochrome apoprotein and chromophore co-ordination that is critical for holophytochrome production that functions in early light-dependent seedling development. Thus, phytochrome-dependent regulation of *SIG2* expression could represent a mechanism for co-ordinating plastid-localized tetrapyrrole synthesis with nuclear-encoded apophytochrome gene expression, which is still not well understood. However, the observed results are distinct from *hy1* and *hy2* mutants, which exhibit elongated hypocotyls and reduced cotyledon expansion under R, FR, B, and W illumination (Neff and Van Volkenburgh, 1994; Parks and Quail, 1993), suggesting that the absence of SIG2 does not lead to a simple phytochrome chromophore deficiency. Indeed, the expression of phytochrome chromophore biosynthesis genes *HY1* and *HY2* is not apparently impacted in *sig2* mutants under FRc (see Supplementary Fig. S3A at *JXB* online); whereas *HY2* expression is reduced by more than 2-fold under Rc in strong *sig2* mutants (Fig. 8). Thus, the *sig2* mutant phenotype is probably due to a lack of substrate, i.e. haem-derived biliverdin (BV) due to a haem deficiency (Woodson *et al.*, 2013) and, in the case of Rc, an additional enzymatic limitation of *sig2* mutants to synthesize the PΦB chromophore. Thus, there is likely to be a chromophore deficiency in *sig2* lines that results in a phenotype similar in some regards, but not identical, to *hy1* and *hy2* phytochrome chromophore-deficient mutants. These latter mutants may have distinct aspects of their phenotypes associated with the accumulation of tetrapyrrole intermediates in the PΦB synthesis pathway.

In addition to being different from phytochrome chromophore-deficient mutants, the phenotypes of *sig2* mutants are different from *CAB UNDEREXPRESSED* (*cue)* mutants, the latter of which exhibit defects in chlorophyll synthesis and/or accumulation similar to *sig2* mutants, but very limited to no impacts on light-dependent control of hypocotyl elongation and cotyledon opening under R, FR, B or W light (Vinti *et al.*, 2005). Furthermore, *cue* mutants exhibit light-dependent and light-independent defects in plastid development (Vinti *et al.*, 2005), unlike *sig2* mutants that exhibit defects that appear to be specific to photomorphogenesis.

Among other mutants impacting light-dependent growth and plastid metabolism, only *laf6* has a somewhat similar phenotype to the strong *sig2* mutants described here. A *laf6* mutant has elevated protoporphyrin IX levels, reduced chlorophyll levels, and elongated hypocotyls specifically under FR light (Møller *et al.*, 2001). *LAF6* encodes an ABC transporter (i.e. AtABC1), which was purported to function as a chloroplast transporter, perhaps transporting tetrapyrroles out of the plastid (Møller *et al.*, 2001). The elevated protoporphyrin IX levels have been postulated to be associated with a reduced phytochrome chromophore pool in *laf6* mutants (Cornah *et al.*, 2003). However, why this would result in a FR-specific defect in hypocotyl elongation, and not also impact Rc-dependent development, is unclear.

*SIG2* expression is regulated by both phyA and phyB in a light-dependent manner (Fig. 1C; see Supplementary Fig. S1 at *JXB* online), whereas SIG2 has a minor impact on *PHYA* and *PHYB* mRNA accumulation (see Supplementary Fig. S2 at *JXB* online). Prior experiments demonstrated a decrease of *SIG2* mRNA accumulation in a *phyA* mutant using microarray analyses (Ma *et al.*, 2001) and in a *phyB* mutant by Northern analysis (Nagashima *et al.*, 2004). Notably, *SIG2* appears to accumulate to a greater level under Rc and W light than under FRc in 7-d-old seedlings (Fig. 2C). Similar up-regulation of *SIG2* in response to FR light was reported using microarray analysis (Ma *et al.*, 2001). Although *SIG2* is up-regulated under Bc in addition to other light conditions, including UV, W, R, and FR (Fig. 2B), hypocotyl length is increased under R and FR, but essentially the same as WT under Bc for strong *sig2* mutants (Fig. 4). However, strong *sig2* mutants are still severely chlorophyll-deficient under Bc (Fig. 5A), which suggests that the B-dependent role of SIG2 includes the regulation of chloroplast development and chlorophyll synthesis.

**Fig. 10.**
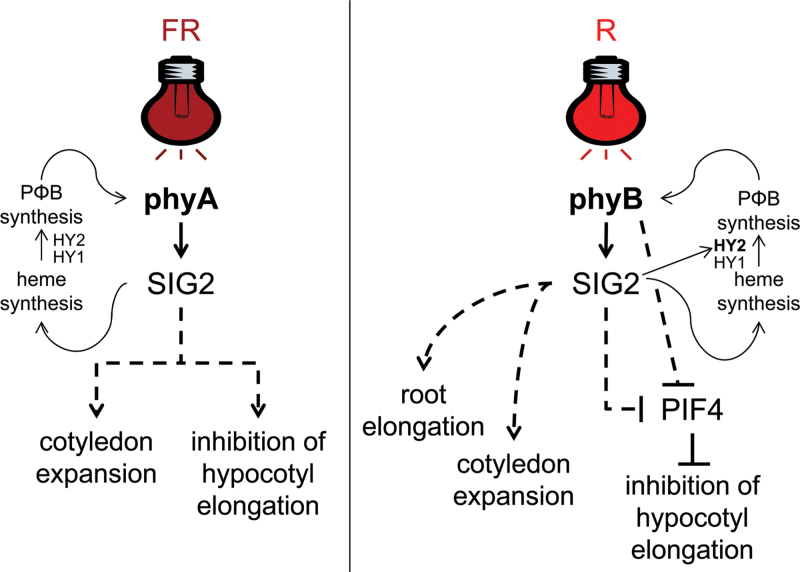
Model depicting the roles of phyA or phyB under FR or R light, respectively, in the regulation of distinct genes and phytochrome-dependent responses. Sigma factor 2 (SIG2), phytochrome-interacting factor 4 (PIF4), phytochrome chromophore phytochromobilin (PΦB), phytochrome chromophore biosynthesis enzymes haem oxygenase (HY1), and phytochromobilin synthase (HY2). Solid lines indicated confirmed relationships, whereas dotted lines represent pathways for which factors are not definitively confirmed or are still unknown. FR, far-red light; R, red light.

The extended hypocotyl phenotype and up-regulation of expression of the *PIF4* gene in Rc-grown *sig2* mutants (Figs 4, 7) are reminiscent of phenotypes observed in *PIF4*-overexpressing lines (Huq and Quail, 2002). In Rc, *PIF4*-overexpressing lines show longer hypocotyls whereas *PIF4* knock-down lines exhibit shorter hypocotyls, suggesting that *PIF4* is a negative regulator of phyB signalling (Huq and Quail, 2002). Up-regulation of *PIF4* in *sig2* mutants may explain, in part, the longer hypocotyl phenotypes observed in *sig2* mutants in Rc. The elongated hypocotyl and down-regulation of expression of *HY2* under Rc are also consistent with phenotypes of the *hy2* mutants (Koornneef *et al.*, 1980). Thus, it is proposed that *SIG2* contributes to phyB signalling by negative transcriptional regulation of *PIF4* and positive regulation of *HY2* under R, which could be a direct regulation or, alternatively, could be the result of reduced levels of holophytochromes due to reduced chromophore levels (Woodson *et al.*, 2013) in the *sig2* mutants (Fig. 10). Notably, expression of *CIPK20* was also identified as down-regulated in a *sig2* mutant under W light (Woodson *et al.*, 2013) and in phytochrome-deficient lines under R and FR light (Oh *et al.*, 2013). Recently, it was demonstrated that CIPK20 contributes to phytochrome-dependent inhibition of hypocotyl elongation under R and FR (Oh *et al.*, 2013). Taken together, these results suggest a potential for CIPK20 to be regulated by phytochromes in a SIG2-dependent manner to impact photomorphogenesis in *Arabidopsis*.

In chloroplasts, two types of RNA polymerase complexes, nuclear-encoded RNA polymerase (NEP) and plastid-encoded RNA polymerase (PEP) perform transcriptional events. Interaction of PEP with sigma factors (SIGs) at the promoters of target genes in chloroplasts is required for the initiation of transcription of many genes (Beardslee *et al.*, 2002; Suzuki *et al.*, 2004). In *Arabidopsis*, PEP also physically interacts with Transcriptionally Active Chromosome protein 12 (pTAC12), also named HEMERA (HMR; Pfalz *et al.*, 2006; Chen *et al.*, 2010), which functions in phytochrome signalling in the nucleus (Chen *et al.*, 2010). HMR has recently been shown to interact physically with phytochromes (Galvão *et al.*, 2012). HMR is essential for the proteolysis of phytochrome, PIF1, and PIF3 in the nucleus, yet is also localized to plastids and has a function in these organelles as nuclear-only *HMR* expression does not fully rescue the *hmr* mutant phenotype (Chen *et al.*, 2010). HMR has been considered an indirect transcriptional regulator for PIF1/PIF3-controlled genes encoding chloroplast proteins (Chen *et al.*, 2010). Since PIF4 shares significant homology with other PIF family members, including PIF1 and PIF3 (Leivar and Quail, 2011), it could be interesting to test whether chloroplast-localized HMR is associated with the SIG2-dependent phenotypes that were observed. Notably, expression of *HMR* does not appear to be impacted by light, although HMR protein accumulates preferentially in light (Chen *et al.*, 2010), which has recently been shown to be due to light-dependent promotion of HMR protein accumulation due to interactions with light-activated phytochromes (Galvão *et al.*, 2012). *SIG2* expression and protein accumulation are both light-dependent (Isono *et al.*, 1997; Privat *et al.*, 2003).

It has been suggested previously that levels of SIG proteins, which are enriched in leaf tissues, could be involved in the regulation of plastid-localized photosynthesis genes in a tissue-specific manner (Isono *et al.*, 1997). Part of the tissue-specific phytochrome responses involve the regulation of genes encoding tissue-enriched factors such as SIG2, CIPK20 and other genes (Oh *et al.*, 2013). Although the expression of *SIG2* is leaf-specific (Isono *et al.*, 1997), a clear impact on hypocotyl and root elongation is seen in *sig2* mutants. These observations correspond to prior findings that light perception in cotyledon or leaf tissue results in a signal(s) that inhibits growth in hypocotyls (Tanaka *et al.*, 2002; Endo *et al.*, 2005; Montgomery, 2009; Warnasooriya and Montgomery, 2009; Oh *et al.*, 2013) and impacts root elongation (Costigan *et al.*, 2011). Although phytochrome-dependent impacts of light on root elongation have been reported previously (Costigan *et al.*, 2011; Dyachok *et al.*, 2011), an indirect impact of aberrant chloroplast development on the inhibition of root elongation that is observed in strong *sig2* mutants cannot summarily be ruled out.

As a result of reciprocal effects of light during de-etiolation, elongation of hypocotyls is inhibited, but cotyledon expansion is enhanced. It has been suggested that these opposite, organ-specific responses can be used as an excellent screening method to distinguish between mutants related to general cell expansion and those specifically related to light-regulated cell expansion (Quail, 2002). In this regard, *sig2* mutants exhibited FR- or Rc-dependent defects in cotyledon expansion, inhibition of hypocotyl elongation, and promotion of root elongation, which differ from mutants showing general defects in seedling growth and development (e.g. dwarf mutants). Therefore, SIG2 most likely contributes to light-signalling events that are vitally important for apposite regulation of distinct aspects of photomorphogenesis.

## Supplementary data

Supplementary data can be found at *JXB* online.

Supplementary Table S1. Additional primer sequences used in this study

Supplementary Fig. S1. Expression of *SIG2* in Dc-grown phytochrome-deficient lines.

Supplementary Fig. S2. Expression of *PHYA* and *PHYB* in *sig2* mutants.

Supplementary Fig. S3. Expression of phytochrome-related genes in *sig2* mutants (A) and expression of *SIG2* in *hy5* mutants (SALK_056405) or *pif4* mutants (SALK_140393) (B) under FRc.

Supplementary Fig. S4. Expression of phytochrome-related genes in *sig2* mutants (A) and expression of *SIG2* in *hy5* (SALK_056405) or *pif4* (SALK_140393) mutants (B) under Rc.

Supplementary Data
