# Supplementary material for: Phytochrome-induced SIG2 expression contributes to photoregulation of phytochrome signalling and photomorphogenesis in Arabidopsis thaliana
Source: J Exp Bot. 2013 Sep 27;64(18):5457–72. doi: 10.1093/jxb/ert308 (PMC3871806; doi:10.1093/jxb/ert308)
Supplement: Supplementary Data [file supp_64_18_5457__index.html]

Phytochrome-induced SIG2 expression contributes to photoregulation of phytochrome signalling and photomorphogenesis in Arabidopsis thaliana — Phytochrome-induced SIG2 expression contributes to photoregulation of phytochrome signalling and photomorphogenesis in Arabidopsis thaliana — Supplementary Data 

# Phytochrome-induced *SIG2* expression contributes to photoregulation of phytochrome signalling and photomorphogenesis in *Arabidopsis thaliana*

## Supplementary Data

Data files

**Files in this Data Supplement:**

- Supplementary Data - Supplementary Data
