# Supplementary material for: Phytochrome-induced SIG2 expression contributes to photoregulation of phytochrome signalling and photomorphogenesis in Arabidopsis thaliana
Source: J Exp Bot. 2013 Sep 27;64(18):5457–72. doi: 10.1093/jxb/ert308 (PMC3871806; doi:10.1093/jxb/ert308)
Supplement: Supplementary Data [file supp_ert308_jexbot106096_file001.pdf]

Phytochrome-induced *SIG2* expression contributes to photoregulation of phytochrome signaling and photomorphogenesis in *Arabidopsis thaliana*

Sookyung Oh[1] and Beronda L. Montgomery[1,2*]

SUPPLEMENTARY DATA

Table S1. Additional primer sequences used in this study

AGI Number	LP or Forward primer sequence (5'-3')	RP or Reverse primer sequence (5'-3')	Purpose
At2g36990 (*SIG6*)	acggctgcgaaccaacaatagg	tgttggtgtctcaatcccactgtct	RT-PCR analysis
At1g09570 (*PHYA*)	gctgtgaccagtcaagatcca	cacctgtcactacaccttccc	qRT-PCR analysis
At2g18790 (*PHYB*)	tgcagttgcaagagagatggt	ttccatccattgatgcagcct	qRT-PCR analysis
At5g11260 (*HY5*)	catcaagcagcgagaggtcatca	aggcttgcatcagcattagaaccac	RT-PCR analysis
At2g43010 (*PIF4*)	gcaatcggtaacaagtcgaaccaa	cgccggtgaactaaatctcaacatc	RT-PCR analysis
At2g26670 (*HY1*)	tcctcaccgaacccacttttcc	tctgccacctttctgccaatca	RT-PCR analysis
At3g09150 (*HY2*)	tttgggttttcaattgggtcatgct	cacatcaccaaaggcgagaaaaact	RT-PCR analysis

Supplementary Figure Legends

Figure S1. Expression of *SIG2* in Dc-grown phytochrome-deficient lines. (A) qRT-PCR analysis was performed using seven-day-old No-0 wild-type (WT), 35S::pBVR3 (35S), and CAB3::pBVR2 (CAB3). (B) qRT-PCR analysis was performed using seven-day-old Col-0 wild-type (WT), *phyA*, *phyB*, and *phyAphyB* mutant seedlings. Plants were grown at 22 ºC on MS medium containing 1% Suc under Dc. Relative expression level compared to *UBC21* is shown (±SD, n=3).

Figure S2. Expression of *PHYA* and *PHYB* in *sig2* mutants. qRT-PCR analysis was performed using seven-day-old Col-0 wild-type (WT) and mutant seedlings grown at 22 ºC on MS-medium containing 1% Suc under FRc (2.5 µmol $m^{-2}s^{-1}$) or Rc (50 µmol m^{-2} s^{-1}). Relative expression level compared to *UBC21* is shown (±SD, n=3).

Figure S3. Expression of phytochrome-related genes in *sig2* mutants (A) and expression of *SIG2* in *hy5* mutants (SALK_056405) or *pif4* mutants (SALK_140393) (B) under FRc. RT-PCR analysis was performed using seven-day-old Col-0 wild-type (WT) and mutant seedlings grown on MS medium containing 1% Suc and 0.7% Phytoblend agar at 22 ºC under FRc (5 µmol m^{-2} s^{-1}). *UBC21* gene was used as an internal control. Results shown are representative of two independent biological replicates.

Figure S4. Expression of phytochrome-related genes in *sig2* mutants (A) and expression of *SIG2* in *hy5* (SALK_056405) or *pif4* (SALK_140393) mutants (B) under Rc. RT-PCR analysis was performed using seven-day-old Col-0 wild-type (WT) and mutant seedlings grown on MS medium containing 1% Suc and 0.7% Phytoblend agar at 22 ºC under Rc (50 µmol $m^{-2}s^{-1}$). *UBC21* gene was used as an internal control. Results shown are representative of two independent biological replicates.

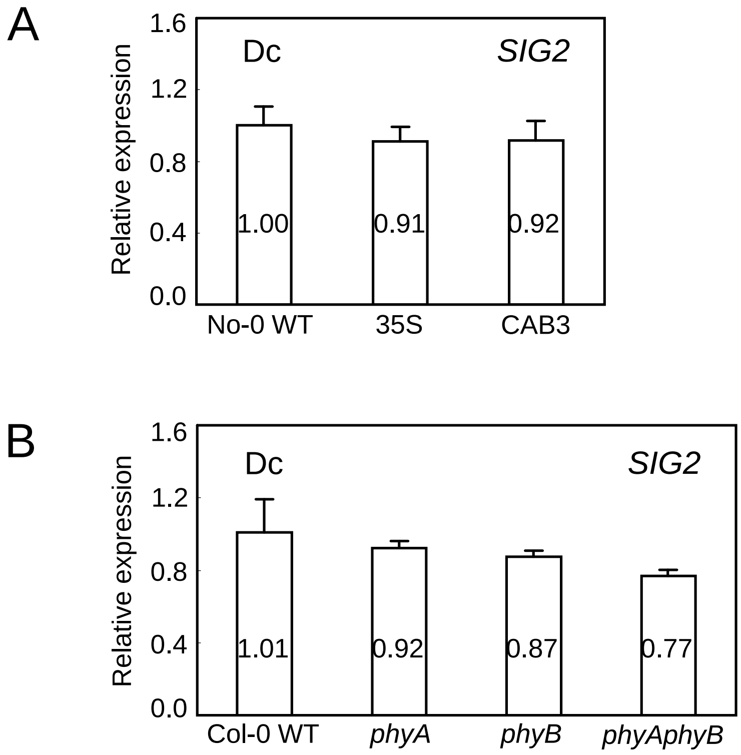

Supplemental Figure S1. Expression of *SIG2* in Dc-grown phytochrome-deficient lines. (A) qRT-PCR analysis was performed using seven-day-old No-0 wild-type (WT), 35S::pBVR3 (35S), and CAB3::pBVR2 (CAB3). (B) qRT-PCR analysis was performed using seven-day-old Col-0 wild-type (WT), *phyA*, *phyB*, and *phyAphyB* mutant seedlings. Plants were grown at 22 ºC on MS medium containing 1% Suc under Dc. Relative expression level compared to *UBC21* is shown (±SD, n=3).

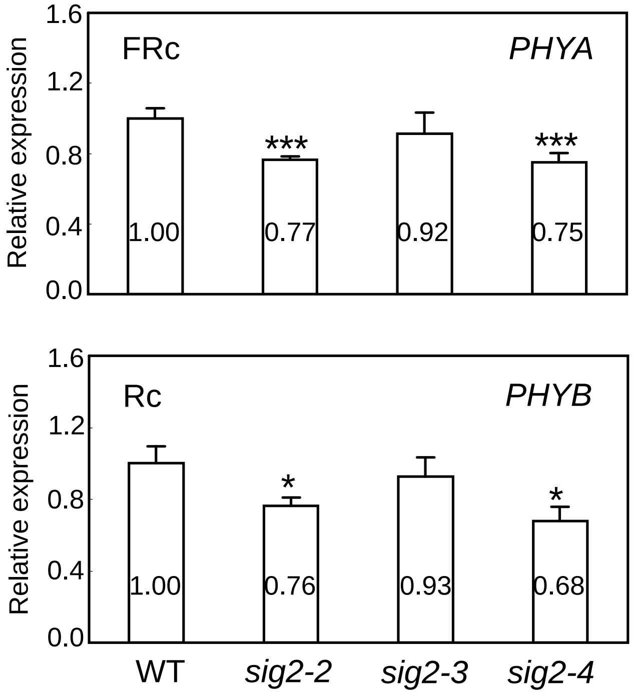

Supplemental Figure S2. Expression of *PHYA* and *PHYB* in *sig2* mutants. qRT-PCR analysis was performed using seven-day-old Col-0 wild-type (WT) and mutant seedlings grown at 22 ºC on MS-medium containing 1% Suc under FRc (2.5 µmol $m^{-2}s^{-1}$) or Rc (50 µmol m^{-2} s^{-1}). Relative expression level compared to *UBC21* is shown (±SD, n=3). Unpaired, two-tailed Student's t test comparing BVR lines or mutant to WT, *, $p<0.05$, ***, $p<0.005$.

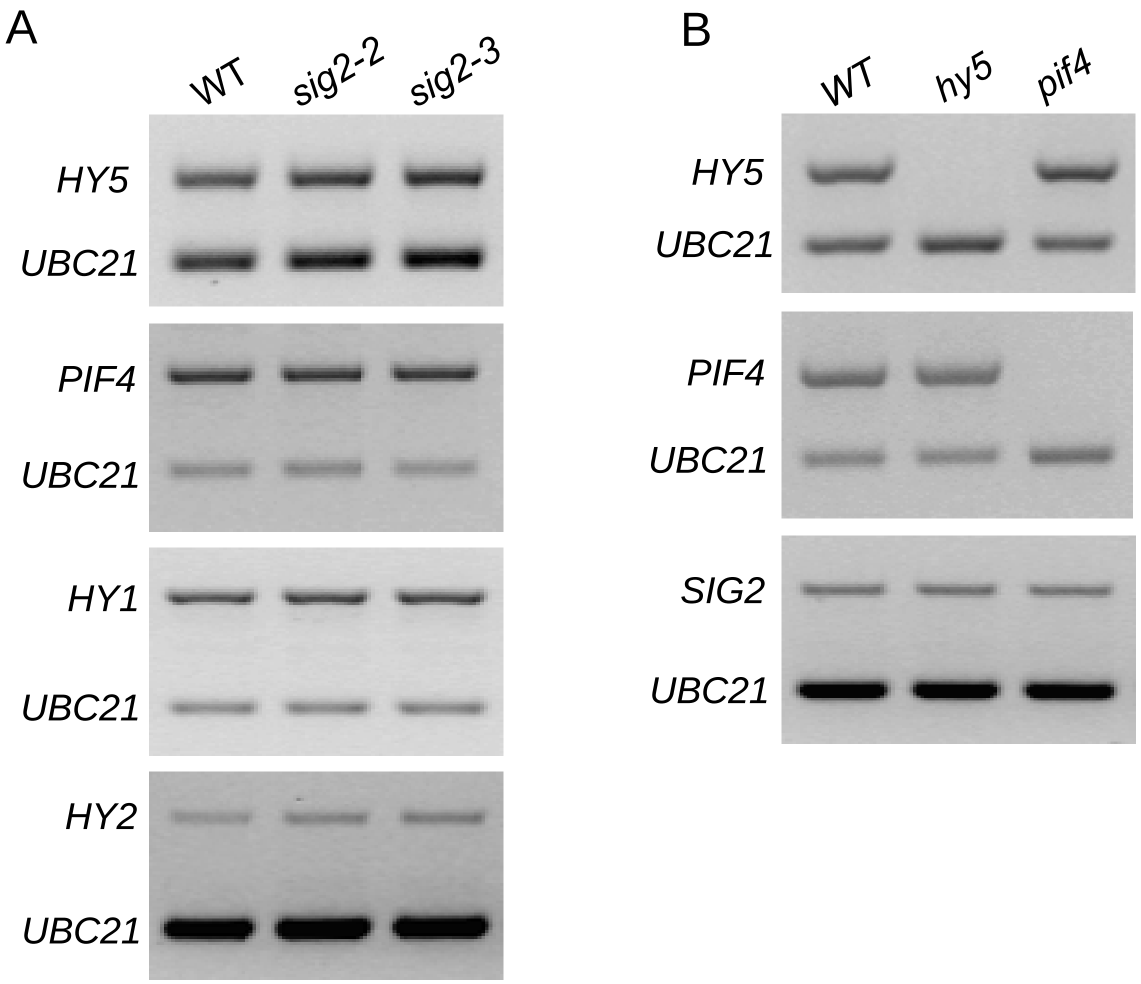

Supplemental Figure S3. Expression of phytochrome-related genes in *sig2* mutants (A) and expression of *SIG2* in *hy5* (SALK_056405) or *pif4* mutants (SALK_140393) (B) under FRc. RT-PCR analysis was performed using seven-day-old Col-0 wild-type (WT) and mutant seedlings grown on MS medium containing 1% Suc and 0.7% Phytoblend agar at 22 ºC under FRc (5 µmol m^{-2} s^{-1}). *UBC21* gene was used as an internal control. Results shown are representative of two independent biological replicates.

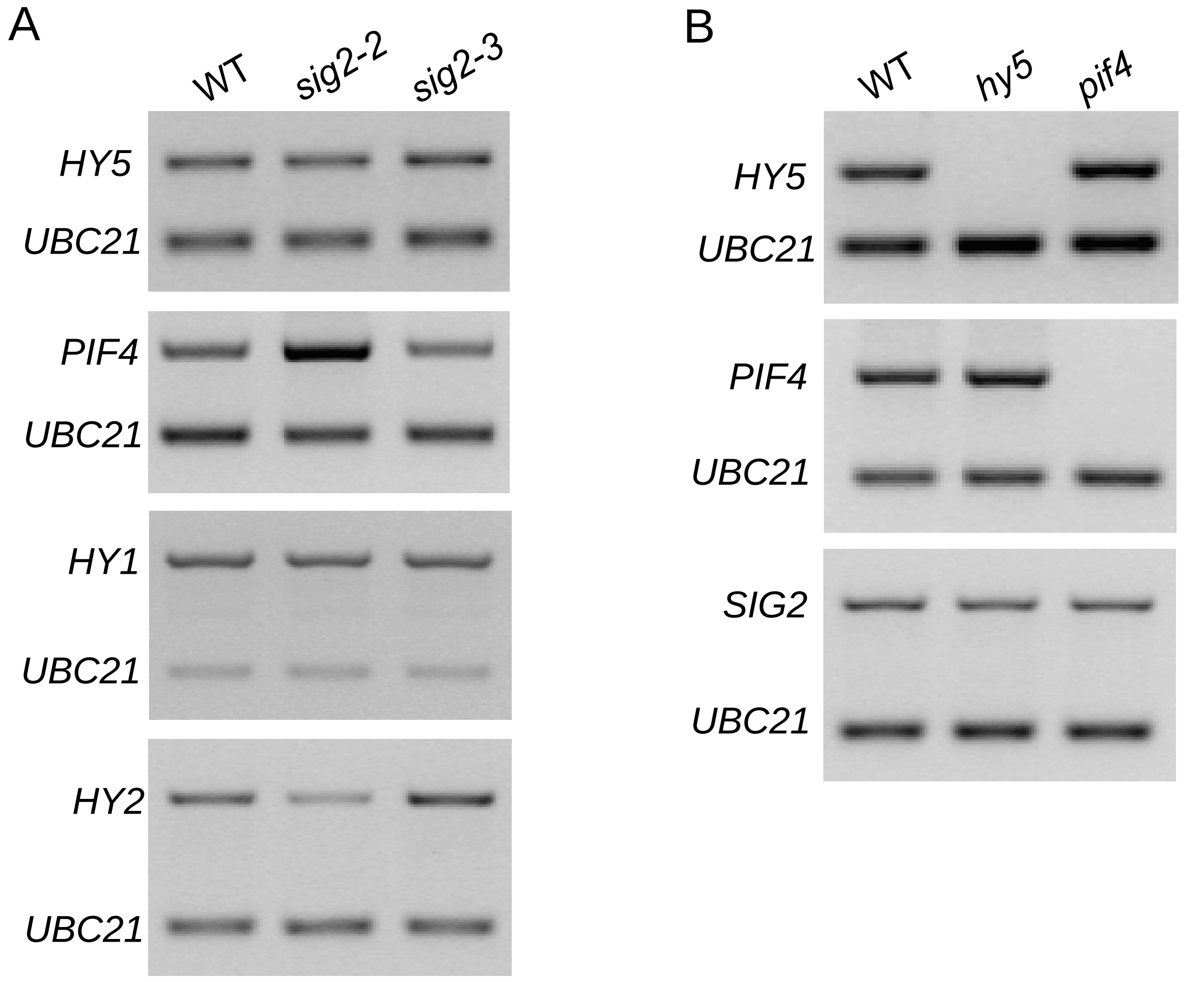

Supplemental Figure S4. Expression of phytochrome-related genes in *sig2* mutants (A) and expression of *SIG2* in *hy5* (SALK_056405) or *pif4* (SALK_140393) mutants (B) under Rc. RT-PCR analysis was performed using seven-day-old Col-0 wild-type (WT) and mutant seedlings grown on MS medium containing 1% Suc and 0.7% Phytoblend agar at 22 ºC under Rc (50 µmol $m^{-2}s^{-1}$). *UBC21* gene was used as an internal control. Results shown are representative of two independent biological replicates.
